# Supplementary material for: Cost-benefit trade-offs of bird activity in apple orchards
Source: PeerJ. 2016 Jun 30;4:e2179. doi: 10.7717/peerj.2179 (PMC4933086; doi:10.7717/peerj.2179)
Supplement: Supplemental Information 1 [file peerj-04-2179-s001.docx]

**Supplementary information**

**Supporting methods 1**

The amount of time each bird spent in the focal tree (from arrival until departure) and its behaviour were recorded in a table using predetermined shorthand notes (only the behaviours actually observed are presented here). Behaviour was assigned to the following five broad categories and recorded every 10 seconds until the bird left the tree: eat apple on tree (before harvest, includes eating apple flowers), forage on tree (actively searching the tree for invertebrates), glean invertebrate from tree (i.e. remove and eat invertebrate from trunk, branch, leaf or fruit), hawk (catch invertebrate in air), and non-feeding activities (groom, fly (flying without catching invertebrates), perch, vocalize, vigilant (head up, bird on ground), interact with another bird, and walk). If no birds were observed in the first 20 minutes of beginning the survey, then the survey was abandoned.

In addition to the focal tree surveys, activity budgets were recorded opportunistically for bird species observed within the orchards (Supporting results 2, Supplementary information). Using binoculars, the behaviour of an individual was observed and recorded every ten seconds for as long as possible. Behaviours included those listed above, as well as: drink water, eat grass flower, eat ground invertebrate (handle invertebrate in beak), forage on ground (head down, searching the ground), eat nectar from apple flower, eat apple on ground, and hover (searching/hunting vertebrates from air, i.e. raptors). Observations were stopped if foliage blocked the view of an individual, or if the bird moved too far away to be seen clearly. There was no way of knowing if the same individual was observed more than once, so data were pooled for each species.

**Supporting results 1**

Species observed in adjacent patches of unmanaged vegetation but not in apple orchards were: white-throated treecreeper (*Cormobates leucophaea*), brown treecreeper (*Climacteris picumnus*), eastern whipbird (*Psophodes olivaceus*), brown thornbill (*Acanthiza pusilla*), little corella (*Cacatua sanguinea*), weebill (*Smicrornis brevirostris*), blue-faced honeyeater (*Entomyzon cyanotis*) and red-rumped parrot (*Psephotus haematonotus*).

**Supporting results 2**

*Activity budgets*

A total of 2596 individual activities were recorded for 34 different species. Average observations lasted for 16 individual activities (i.e. 160 seconds) (range: 3-77 individual activities/ 30-770 seconds). Only observations lasting longer than 50 individual activities were included for analysis (Figure S1, Supplementary information).
