# Supplementary material for: Cost-benefit trade-offs of bird activity in apple orchards
Source: PeerJ. 2016 Jun 30;4:e2179. doi: 10.7717/peerj.2179 (PMC4933086; doi:10.7717/peerj.2179)
Supplement: Table S1 — Feeding types categorised as: O, omnivore; H, herbivore; I, insectivore; G, granivore; and C, carnivore. Introduced species are listed in bold. Asterisks denote which species were observed in particular survey periods. [file peerj-04-2179-s002.docx]

**Table S1. Bird species found in apple orchards over the entire season.** Feeding types categorised as: O = omnivore, H = herbivore, I = insectivore, G = granivore, and C = carnivore. Introduced species are listed in bold. Asterisks denote which species were observed in particular survey periods.

| **Common name** | **Scientific name** | **Feeding type** | **Full bloom** | **Early fruit set** | **Harvest** | **Post-harvest** |
| --- | --- | --- | --- | --- | --- | --- |
| Australian king parrot | *Alisterus scapularis* | H |  |  | * |  |
| Australian magpie | *Cracticus tibicen* | O | * | * | * | * |
| Australian raven | *Corvus coronoides* | O | * | * | * | * |
| Australian white ibis | *Threskiornis moluccus* | O |  | * |  | * |
| Australian wood duck | *Chenonetta jubata* | H | * |  |  |  |
| Black-aced cuckoo-shrike | *Coracina novaehollandiae* | I | * | * |  |  |
| Black-shouldered kite | *Elanus axillaris* | C | * | * |  |  |
| Brown falcon | *Falco berigora* | C | * |  |  | * |
| Crimson rosella | *Platycercus elegans* | H | * | * | * | * |
| Eastern rosella | *Platycercus eximius* | H | * | * | * |  |
| Eastern spinebill | *Acanthorhynchus tenuirostris* | O | * | * |  |  |
| Eastern yellow robin | *Eopsaltria australis* | I |  |  |  |  |
| **European blackbird** | *Turdus merula* | O | * | * | * | * |
| **European goldfinch** | *Carduelis carduelis* | G | * | * | * |  |
| Galah | *Eolophus roseicapilla* | G | * |  |  |  |
| Golden whistler | *Pachycephala pectoralis* | I |  |  |  | * |
| Grey currawong | *Strepera versicolor* | O | * | * |  |  |
| Grey fantail | *Rhipidura albiscapa* | I | * | * | * | * |
| Grey shrike-thrush | *Colluricincla harmonica* | I | * | * |  |  |
| **House sparrow** | *Passer domesticus* | G | * | * |  |  |
| Laughing kookaburra | *Dacelo novaeguineae* | C | * |  |  | * |
| Magpie lark | *Grallina cyanoleuca* | I | * |  |  |  |
| Nankeen Kestrel | *Falco cenchroides* | C | * |  |  |  |
| New Holland honeyeater | *Phylidonyris novaehollandiae* | O | * |  |  |  |
| Pied currawong | *Strepera graculina* | O |  |  |  | * |
| Red wattlebird | *Anthochaera carunculata* | O | * |  |  | * |
| Red-browed finch | *Neochmia temporalis* | G | * |  | * |  |
| Red-capped robin | *Petroica goodenovii* | I | * | * |  | * |
| Rufous whistler | *Pachycephala rufiventris* | I | * |  |  |  |
| Satin bowerbird | *Ptilonorhynchus violaceus* | O | * | * |  | * |
| Scarlet robin | *Petroica boodang* | I | * |  |  | * |
| Silvereye | *Zosterops lateralis* | O | * | * | * | * |
| Striated thornbill | *Acanthiza lineata* | I |  |  | * | * |
| Superb fairy-wren | *Malurus cyaneus* | I | * | * | * | * |
| Welcome swallow | *Hirundo neoxena* | I | * |  |  |  |
| White-faced heron | *Egretta novaehollandiae* | C | * |  |  |  |
| Willie wagtail | *Rhipidura leucophrys* | I | * |  | * | * |
| Yellow-faced honeyeater | *Lichenostomus chrysops* | O | * | * | * | * |
| Yellow-rumped thornbill | *Acanthiza chrysorrhoa* | I | * | * | * | * |
