# Supplementary figures and images for: Cost-benefit trade-offs of bird activity in apple orchards
Source: PeerJ. 2016 Jun 30;4:e2179. doi: 10.7717/peerj.2179 (PMC4933086; doi:10.7717/peerj.2179)

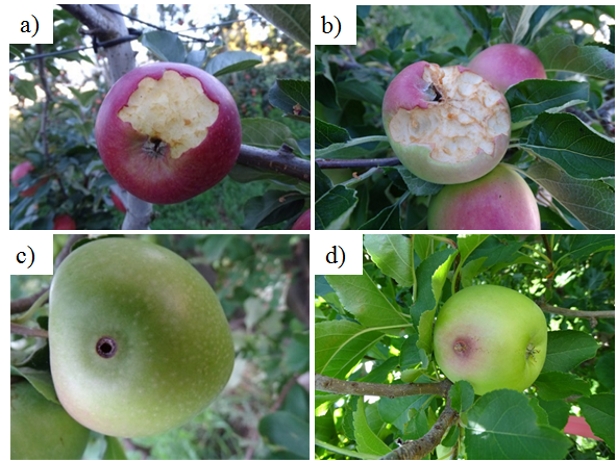

Supplement: Figure S3 — a + b: bird damage, c + d: insect damage. [file peerj-04-2179-s004.jpg]

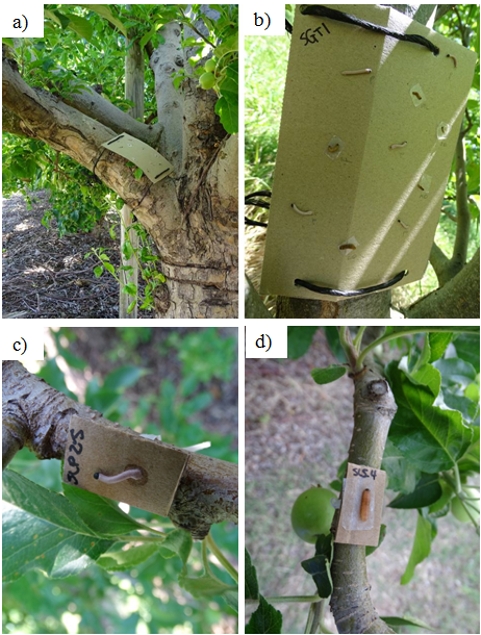

Supplement: Figure S4 — (A) and (B) Plasticine and real codling moth larvae attached to cardboard trays to be monitored by Reconyx HC500 remote motion-sensor cameras, (C) Plasticine codling moth larvae attached to apple tree branches near apple clusters, and d) real codling moth larvae attached to apple tree branches near apple clusters. [file peerj-04-2179-s005.jpg]

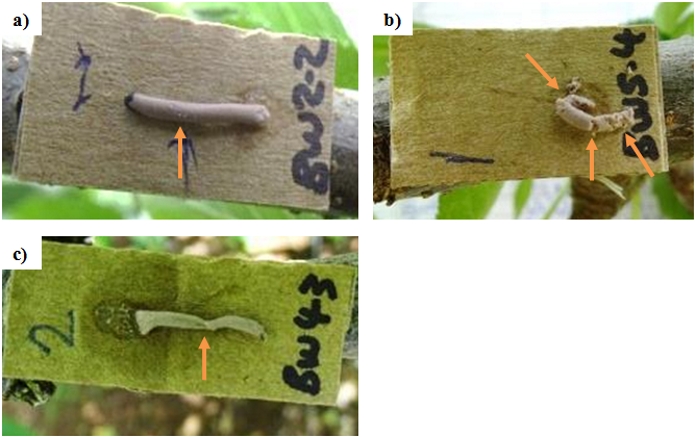

Supplement: Figure S5 — (A) Insect damage—pinpricks; (B); insect damage—chew; (C) bird damage—beak mark. [file peerj-04-2179-s006.jpg]

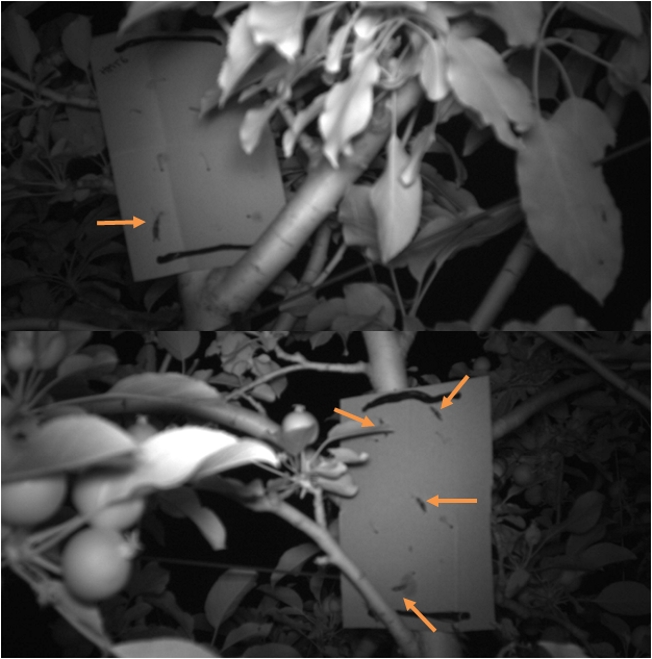

Supplement: Figure S1 [file peerj-04-2179-s009.jpg]
